# The ionophore antibiotic gramicidin A inhibits pancreatic cancer stem cells associated with CD47 down-regulation

**DOI:** 10.1186/s12935-019-0862-6

**Published:** 2019-05-22

**Authors:** Rui-Qi Wang, Jing Geng, Wei-Jin Sheng, Xiu-Jun Liu, Min Jiang, Yong-Su Zhen

**Affiliations:** NHC Key Laboratory of Biotechnology of Antibiotics, Laboratory of Oncology, Institute of Medicinal Biotechnology, Chinese Academy of Medical Sciences and Peking Union Medical College, 1 Tiantan Xili, Beijing, 100050 China

**Keywords:** Gramicidin A, Ionophore antibiotic, Cancer stem cell, CD47

## Abstract

**Background:**

Pancreatic cancer stem cells (CSCs), a special population of cells, renew themselves infinitely and resist to various treatment. Gramicidin A (GrA), an ionophore antibiotic derived from microorganism, can form channels across the cell membrane and disrupt cellular ionic homeostasis, leading to cell dysfunction and death. As reported, the ionophore antibiotic salinomycin (Sal) has been proved to kill CSCs effectively. Whether GrA owns the potential as a therapeutic drug for CSCs still remains unknown. This study investigated the effect of GrA on pancreatic CSCs and the mechanism.

**Methods:**

Tumorsphere formation assay was performed to assess pancreatic CSCs self-renewal potential. In vitro hemolysis assay was determined to test the borderline concentration of GrA. CCK-8 assay was used to detect pancreatic cancer cell proliferation capability. Flow cytometry was performed to detect cell apoptosis and mitochondrial membrane potential. Scanning and transmission electron microscopy was used to observe ultrastructural morphological changes on cell membrane surface and mitochondria, respectively. Western blot analysis was used to determine relative protein expression levels. Immunofluorescence staining was performed to observe CD47 re-distribution.

**Results:**

GrA at 0.05 μM caused tumorspheres disintegration and decrease in number of pancreatic cancer BxPC-3 and MIA PaCa-2 cells. GrA and Sal both inhibited cancer cell proliferation. The IC50 values of GrA and Sal for BxPC-3 cells were 0.025 μM and 0.363 μM; while for MIA PaCa-2 cells were 0.032 μM and 0.163 μM, respectively. Compared on equal concentrations, the efficacy of GrA was stronger than that of Sal. GrA at 0.1 μM or lower did not cause hemolysis. GrA induced ultrastructural changes, such as the decrease of microvilli-like protrusions on cell surface membrane and the swelling of mitochondria. GrA down-regulated the expression levels of CD133, CD44, and CD47; in addition, CD47 re-distribution was observed on cell surface. Moreover, GrA showed synergism with gemcitabine in suppressing cancer cell proliferation.

**Conclusions:**

The study found that GrA was highly active against pancreatic CSCs. It indicates that GrA exerts inhibitory effects against pancreatic CSCs associated with CD47 down-regulation, implying that GrA might play a positive role in modulating the interaction between macrophages and tumor cells.

## Background

Pancreatic cancer is a devastating disease associated with universally poor prognosis, highlighted by the close parallel between disease incidence and mortality [1]. Pancreatic ductal adenocarcinoma (PDAC), the major histological subtype comprising 90% of all pancreatic cancers, is considered one of the deadliest human cancers, with 1–5%-year survival rates (~ 6-month median survival duration) despite therapy [2]. In the last 40 years, survival rates for patients with PDAC have remained unchanged, while patients with a variety of other cancer types have seen remarkable increases in survival. Recent studies demonstrate that self-renewing cancer stem cells (CSCs) contribute to metastatic dissemination and therapy failure, consequently causing high mortality of PDAC.

Pancreatic CSCs are considered to be a special population of cells with characteristic cell surface expression profile discovered in a plethora of human pancreatic malignancies, which are able to renew themselves infinitely, making multilineage differentiation and resisting to electromagnetic and chemical insults. The most accepted pancreatic CSC markers were CD133, CD44, CD24, CXCR4 and ALDH1 [3, 4]. As reported, pancreatic CD133^+^ CSCs (not on other nonmalignant cells in the pancreas) displayed an up-regulated CD47 which was considered as the “don’t eat me” signal protecting CSCs from macrophage-mediated phagocytosis [5]. Many attempts at finding novel pancreatic CSC inhibitors and agents re-sensitizing CSC to current cytotoxic therapies have been exploited. The ideal therapeutic approaches to CSC elimination include not only killing cancer cells of the primary tumor, but also obtaining a durable clinical response with the prevention of tumor recurrence and metastasis [6]. There are several potential targets to inhibit CSCs including pancreatic CSC-specific surface markers [7], stem-cell specific signaling pathways (e.g. Notch, Wnt and Hedgehog [6]), resistance mechanisms, the components of CSC niche [8], and mitochondria dysfunction [9, 10]. Based on the targets, there are some new drug strategies including salinomycin (Sal), sorafenib [11] and metformin [12]. It is worth mentioning that Sal, an ionophore antibiotic isolated from *Streptomyces albus*, selectively targets tumorsphere initiation and the growth of CD133^+^ PDAC CSCs and displays a synergistic effect when combined with gemcitabine (GEM) [13]. However, very few CSC-targeting drugs are currently available for clinical use; there is an urgent need for investigation of novel drugs with improved efficacy and reduced toxicity in CSC-targeting therapy.

Gramicidin A (GrA), similar to Sal as an ionophore antibiotic, is a hydrophobic linear pentadecapeptide derived by the microbe *Bacillus brevis* and produced by fermentation technology. As a model membrane protein [14], the GrA ion channel, a formation of head-to-head dimers by hydrogen bonding across the cell membrane, renders biological membrane permeable to specific monovalent cations, leading to disruption of cellular ionic homeostasis and cell dysfunction. Notably, it appears that the ion channel-forming effect might give a new antitumor mission for the old antimicrobial antibiotic [15]. Sal has been proved to kill human CSCs efficiently for several years [16–18]. However, studies on the antitumor effect of GrA were quite limited. As recently reported, GrA combined with curcumin can induce cell apoptosis and overcome multidrug resistance in human breast adenocarcinoma cells [19]. In renal cell carcinoma, GrA might possess the qualities of a cytotoxic and antiangiogenic drug in vitro and in vivo [20, 21]. Meanwhile, GrA-inspired peptides were designed for cancer nanotherapeutics and induced mitochondrial depolarization and apoptotic cell death in breast cancer cell line [22]. Whether GrA owns the potential as a therapeutic drug for CSCs and PDAC still remains unknown so far [23]. Our study aims at the action of GrA on pancreatic CSCs and its mechanism.

## Methods

### Cell culture

Human PDAC cell lines BxPC-3 and MIA PaCa-2 were obtained from American Tissue Culture Collection (ATCC). Mouse macrophage RAW264.7 cells and human monocyte THP-1 cells were obtained from Cell Research Center, Institute of Basic Medical Sciences, Peking Union Medical College. The cells were authenticated, stored according to the supplier’s instructions. MIA PaCa-2 and RAW264.7 cells were cultured in Dulbecco’s Modified Eagle’s Medium (DMEM) or BxPC-3 and THP-1 cells in RPMI 1640 (Hyclone, Logan, UT) supplemented with 100 IU penicillin, 100 μg/mL streptomycin and 10% fetal bovine serum (Gibco BRL, Grand Island, NY, USA) in a humidified atmosphere of 5% CO_2_ and 95% air at 37 °C.

### Chemicals and antibodies

GrA (ALX-350-233) was purchased from ENZO life sciences. Sal (46729) and GEM (Y0000675) were purchased from Sigma-Aldrich. A variety of antibodies used in this study included rabbit anti-CD133 (ab19898), mouse anti-VDAC (ab14734) from Abcam (Cambridge, MA); mouse anti-CD44 (156-3C11, #3570), rabbit anti-c-Myc (D84C12, #5605), rabbit anti-GAPDH (14C10, #2118), rabbit anti-PARP (#9542) and rabbit anti-SIRPα (D6I3 M, #13379) from Cell Signaling Technology (Danvers, MA); mouse anti-ALDH1 (60171-1-Ig) and rabbit anti-CD68 (28058-1-AP) from Proteintech (Rosemont, IL); mouse anti-CD47 (MA5-11895) from Thermo Fisher Scientific (Rockford, IL) as primary antibodies. HRP-conjugated anti-Mouse IgG (ZDR-5307), and HRP-conjugated anti-Rabbit IgG (ZDR-5306) from Zhongshan Golden Bridge Biotechnology, Beijing, China were used as secondary antibodies.

### Proliferation assay

About 5000 cells were seeded in 96-well plate and incubated in 37 °C, 5% CO_2_. After 24 h, the cells were treated with various concentrations of GrA, Sal and GEM in media with 10% FBS for an appropriate time (e.g., 12, 24 or 48 h). Each concentration was triplicate. After removal of supernatants, cell viability was assessed by Cell Counting Kit 8 (CCK-8) assay, which was added 100 μL tenfold CCK-8 solution (Dojindo, Cat. CK04, Tokyo, Japan) and incubated for 1–4 h in the 37 °C following by measurement of the absorbance at 450 nm using a microplate reader. Percent survival for each sample was calculated as 100 × [(OD450 of sample − OD450 of negative control)/(OD450 of positive control − OD450 of negative control)]. CDI (coefficient of drug interaction) value was used as a standard to evaluate the synergistically inhibitory effect of drug combinations, which was calculated as follows: CDI = AB/(A × B). According to the absorbance of each group, A or B was the ratio of the single treatment to control, AB was the ratio of the combination to control. CDI value less than, equal to or greater than 1 indicates that the drugs are synergistic, additive or antagonistic, respectively.

### Tumorsphere formation assay

BxPC-3 and MIA PaCa-2 cells were dissociated into single cells with 2.5% trypsin following by PBS pH 7.2 washing twice. After washing and centrifugation to remove trypsin, 50 cells/well were seeded in serum-free DMEM/F-12 (1:1 ratio) media with B-27 supplement (Gibco, Cat. 17504044, Grand Island, NY, USA), 10 ng/mL bFGF (Living Biotechnologies, Beijing, China), 20 ng/mL EGF (Living Biotechnologies, Beijing, China) in ultra-low attachment 96-well plates (Corning, NY, USA) to form tumorspheres. After 3 days, the cells were observed on the formation of small tumorspheres and treated with compounds. The cells were further incubated with 7 days. The images were collected with optical microscope.

### In vitro hemolysis assay

Blood sample was collected from a 1 kg body-weighted, healthy rabbit (*Oryctolagus cuniculus*) ear vein, and transferred to a centrifuge tube that contained anticoagulant. After centrifuged at 1500 rpm for 15 min, the supernatant was removed. After gently washed with cold PBS for 3 times and adjusted to 2% (v/v) suspension, the erythrocytes were treated with different concentrations of GrA for 1 h at 37 °C. For hemolysis calibration graph, 1% Triton X-100 solution was used as a positive control, and PBS served as a negative control. Samples were artificially prepared by performing 5 Triton X-100 1:10 serial dilutions ranging from 0.0001 to 1%. The amounts of hemoglobin released from the lysed erythrocytes were evaluated by measuring the absorbance at 570 nm. Percent hemolysis for each sample was calculated as 100 × [(OD570 of sample − OD570 of negative control)/(OD570 of positive control − OD570 of negative control)]. All samples were analyzed in triplicate for each experiment, and the averages of three independent experiments ± standard deviations (SD) are presented.

### Apoptosis detection by flow cytometry

According to the manufacturer’s instructions of Annexin V-FITC Apoptosis Detection Kit (Dojindo, Cat. AD10, Tokyo, Japan), adherent pancreatic cancer cells in plates were washed with cold sterile PBS twice. After removal of supernatants, cells were detached with trypsin (at dilution 1:2 to avoid damages on cellular membrane proteins) for appropriate time. The digestion was stopped with culture medium. Cells were pelleted by centrifugation (1000 rpm 3 min at RT) and then washed twice. Tenfold diluted Annexin V Binding Solution was added to re-suspend the cells (final cell concentration of 1 × 10^6^ cells/mL). 5 μL of Annexin V, FITC conjugate and 5 μL of PI solution was successively added to 100 μL cell suspension per sample. The mixed solution was incubated for 15 min at RT with protection from light and then diluted by 400 μL of tenfold diluted Annexin V binding solution per sample before applied to flow cytometry (BD Biosciences, Accuri C6, USA).

### Western blot analysis

After 24 h of culture, the cells were washed twice in cold sterile PBS. Next, cells were scraped, and pelleted by centrifugation (500 rpm 5 min at 4 °C). Proteins were extracted by using RAPI lysis buffer (Beyotime, Haimen, China) in the presence of a protease inhibitor cocktail (Sigma-Aldrich, Cat. P8340, St. Louis, MO) on ice for 1 h. The protein concentration was quantified using BCA Protein Assay Kit (Thermo Scientific, Cat. 23227, Rockford, IL). 20 mg of the protein samples were solubilized in a sample buffer (Solarbio, Cat. P1041, Beijing, China) and heated for 5 min at 95 °C; and were separated through electrophoresis on SDS-PAGE gel under reducing conditions. BluePlus IV Protein Marker (10–180 kDa) (TransGen Biotech, Beijing, China) was used for presenting the protein molecular weights. Subsequently, the resolved proteins were transferred into PVDF sheets following by saturation of the membranes in TBST buffer containing 10% non-fat milk for 1 h at RT. Next, membranes were incubated with respective primary antibodies (CD133, CD44, c-Myc, VDAC, PARP, SIRPα and CD68 at dilution of 1:1000; CD47 at dilution of 1:50; ALDH1 at dilution of 1:10,000; GAPDH at dilution of 1:2000, respectively) in a solution of 3% BSA in TBST buffer, pH 7.6, overnight at 4 °C with gentle shaking. Then, membranes were washed 3 times for 10 min following by incubation with secondary antibody (at dilution 1:5000) for 1 h at RT. Signals were developed using the enhanced chemiluminescence assay (Millipore, Cat. WBKLS0500, Billerica, MA) and quantified by AlphaView SA. The protein content was normalized to GAPDH for quantification of chemiluminescence density.

### Flow cytometric detection of mitochondrial membrane potential

Cell mitochondrial membrane potential was measured by following the manufacturer’s instructions (Solarbio, Cat. M8650, Beijing, China). After corresponding treatment of drugs, cells were harvested and suspended with complete medium. JC-1 working solution was prepared before adding to the cell suspension. Cell suspensions were gently mixed with JC-1 working solution and incubated at 37 °C for 20 min. After staining, suspensions were centrifuged at 600×*g* for 4 min at 4 °C to remove supernatant, and cells were gently resuspended in JC-1 staining buffer solution. After washing at least 4 times to remove the remained JC-1, cells were resuspended in JC-1 staining buffer solution and were submitted to flow cytometry (BD Biosciences, FACSCalibur, USA) for measurement of mitochondrial membrane potential. The percentage of cells with green fluorescence and those with red fluorescence was collected.

### Immunofluorescence staining

Immunocytochemistry was performed as described by Zheng et al. [24]. Cells were grown on coverslips with polylysine pre-treated. After treated with the tested agents, cells were washed in cold PBS and fixed in 4% paraformaldehyde. Next, cell membrane was permeabilized in blocking buffer (PBS, pH 7.4, 5% FBS and 0.1% NaN_3_) with 0.1–0.2% saponin (Sigma-Aldrich, St. Louis, MO) for 0.5–1 h at RT. Nonspecific binding sites were blocked by incubation in PBS with 5% FBS and 0.1% NaN_3_ in a humidity chamber for 1 h at RT. Cells were incubated with anti-CD47 primary antibody in blocking buffer for 2 h. After incubation, cells were rinsed twice for 5 min in PBS with gentle shaking and incubated with secondary antibody in blocking buffer for 1 h at RT. Cells were washed 6 times again in PBS with gentle shaking. Then, the coverslips were mounted with anti-fade mountant with DAPI (Invitrogen, Cat. P36962, Rockford, IL) and kept under dark condition at RT. The images were collected by Zeiss 710 confocal laser microscope.

### Scanning/transmission electron microscopy (SEM/TEM)

After exposure to GrA or Sal for 24 h at 37 °C, the external morphological changes in pancreatic cancer cells were observed using SEM and the ultrastructural changes in pancreatic cancer cells were observed using TEM. For the preparation of pancreatic cancer cells for SEM and TEM, cells were collected and fixed with 2.5% glutaraldehyde (Solarbio, Cat. P1126, Beijing, China) at 4 °C overnight. Fixed cells were rinsed with PBS and sterile water followed by dehydration through an ethanol gradient. After critical point drying, the samples were coated with gold and electron micrographs were obtained using the Hitachi SU8020 SEM (Tokyo, Japan). Similarly, for TEM, cells were collected as mentioned above, and the samples were embedded in Epon and sectioned with an ultramicrotome. Ultrastructural changes in the cells were observed using the Hitachi JEM-1200EX TEM (Tokyo, Japan).

### Statistical analysis

Experimental data were calculated and analyzed with Microsoft Excel or GraphPad Prism 5 software, which were presented as mean ± SD. Unpaired Student’s t tests (two-sided tests) were used. Statistical analyses were carried out prior to normalization of data. P < 0.05 was considered statistically significant.

## Results

### Gramicidin A inhibited the growth of pancreatic cancer stem cells

**Fig. 1:**
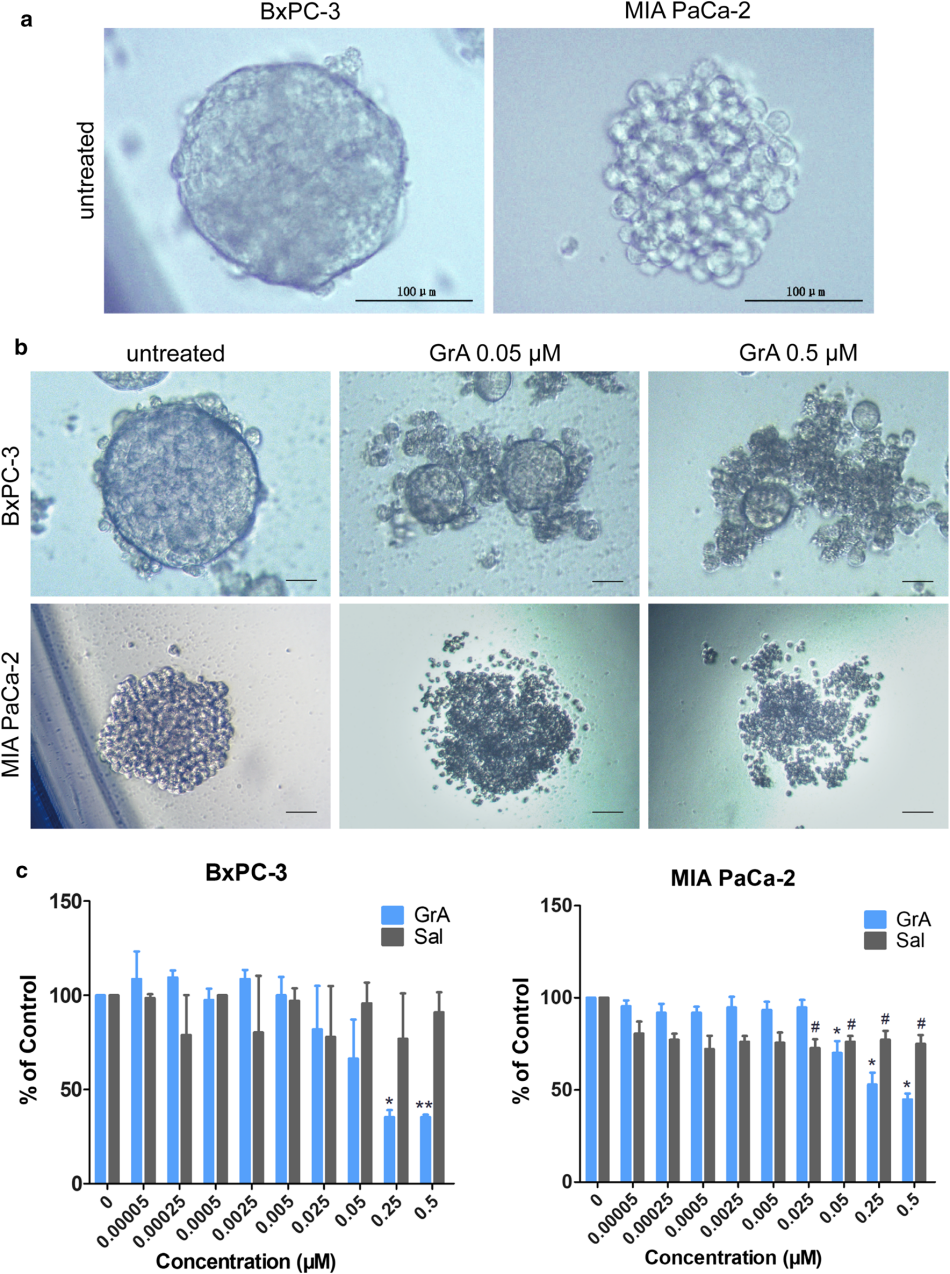
Tumorsphere formation assay. **a** Representative images of tumor spheres in BxPC-3 and MIA PaCa-2 cells obtained before treatment (scale bar: 100 μm). **b** Representative images of BxPC-3 and MIA PaCa-2 tumorspheres obtained after the treatment with the indicated concentrations of GrA for 7 days (scale bar: 50 μm). **c** Statistical analysis of the number (% of control) of BxPC-3 and MIA PaCa-2 tumorspheres treated with GrA or Sal for 7 days, tumorsphere numbers were presented as mean ± SD from 3 replicated wells. *P < 0.05, **P < 0.01, ^#^P < 0.05 vs control

Tumorsphere formation assay is the gold standard assay to assess CSCs self-renewal potential in vitro. When cells are plated at low density in serum-free, non-adherent conditions, only the cells endowed with self-renewal capability are able to survive and proliferate, forming tridimensional clusters/spheres. Those enriched CSCs from tumorsphere assay are usually employed to test the effect of several drugs or certain stimulating factors. In the control well, the tumorsphere from BxPC-3 cells showed a round sphere with a clean edge, and the cells were compacted in the tumorsphere. Compared with BxPC-3 cells, MIA PaCa-2 cells formed a loose and fragile tumorsphere (Fig. 1a). After treated with GrA, the clean edges of tumorspheres in BxPC-3 were broken and the cells turned unconsolidated. As the concentration of GrA increased, the tumorspheres shrunk and the cells disintegrated. At higher concentration, the MIA PaCa-2 tumorspheres also shrunk and fragmentated (Fig. 1b). As shown in Fig. 1c, GrA was highly effective in killing pancreatic CSC in BxPC-3 and MIA PaCa-2 cells; in addition, GrA was more potent than Sal at equal molar concentration.

### Borderline concentration of gramicidin A that caused no hemolysis

**Fig. 2:**
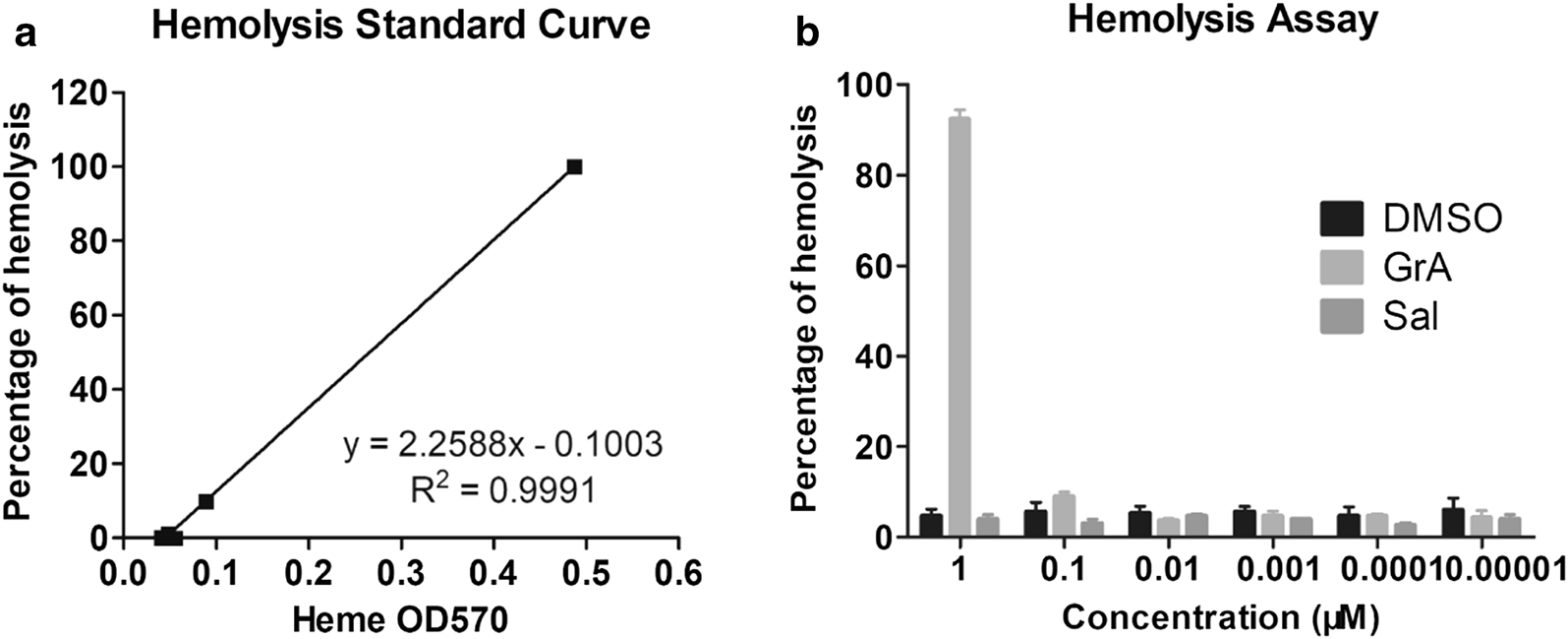
In vitro hemolysis assay of GrA. **a** The calibration graph was prepared by plotting heme OD570 against percentage of hemolysis. **b** Rabbit blood samples were treated separately by GrA, Sal and vehicle (DMSO) in a concentration range of 0.00001–1 μM. OD570 values were detected and then converted to percentage of hemolysis according to calibration graph

GrA has been found to cause hemolysis which becomes one of the barriers for clinical use. In order to detect the borderline concentration of GrA that did not cause hemolysis, the in vitro hemolysis assay using rabbit blood sample was performed. In Fig. 2a, a hemolysis calibration graph plotting showed a hemolysis standard curve which indicated heme OD570 values against the percentage of hemolysis. As shown, GrA at 0.1 μM or lower concentrations did not induce rabbit blood hemolysis, and the OD570 values were close to those of vehicle group (Fig. 2b). It means that GrA at the concentration of 0.1 μM or lower could be relatively safe if used in tumor treatment. Based on this determination, 0.1 μM of GrA was considered to be the borderline concentration that caused no hemolysis and applied in all the following experiments of this study.

### Gramicidin A inhibited cell proliferation and induced apoptosis

**Fig. 3:**
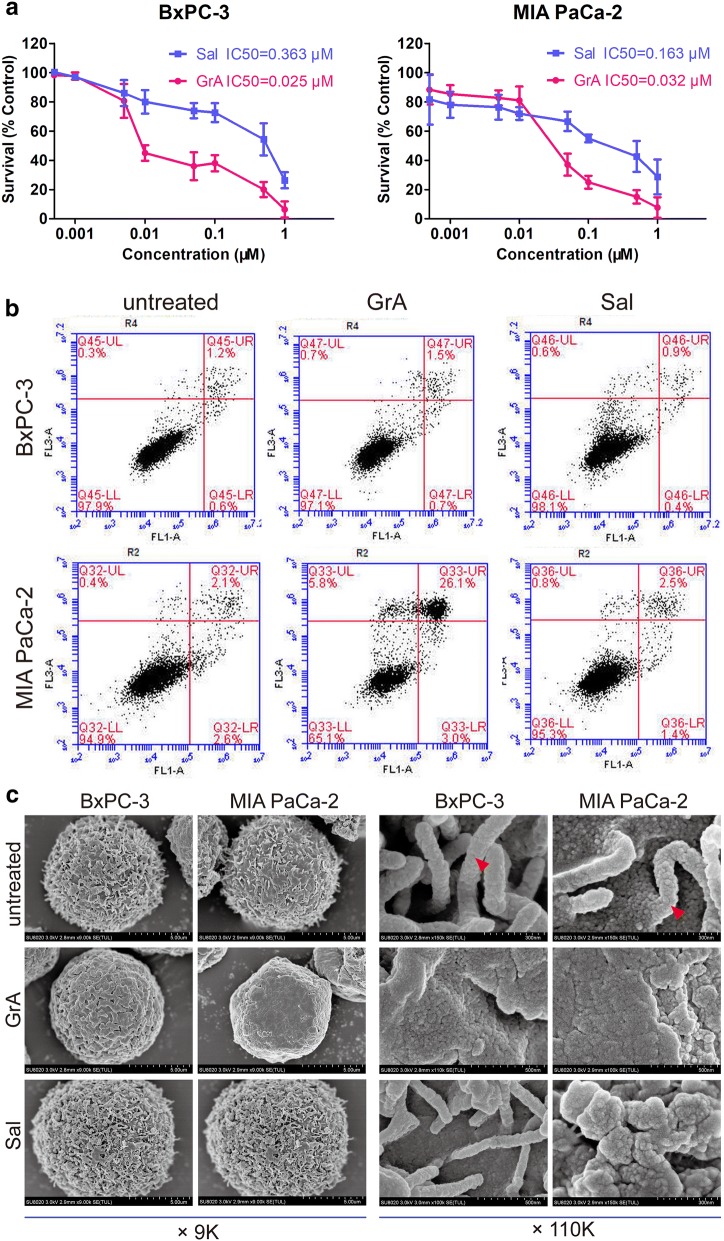
In vitro cytotoxic effects of GrA and Sal on BxPC-3 and MIA PaCa-2 cells. **a** Viability of cultured BxPC-3 and MIA PaCa-2 cells treated with appropriate concentrations of GrA or Sal for 48 h by CCK-8 assay. Data of each concentration were from 3 replicated wells. **b** Flow cytometry results detecting apoptosis in BxPC-3 and MIA PaCa-2 cells were shown. Cells were treated with 0.1 μM GrA or Sal for 24 h. **c** SEM images presenting surface morphological changes of BxPC-3 and MIA PaCa-2 cells treated with 0.05 μM GrA or Sal, on which the red arrow indicates microvilli-like protrusions on cell membrane surface. Scale bar: 5 μm (left), 500 nm (right)

A proliferation assay was applied to detect the cytotoxicity of GrA and Sal, both ionophore antibiotics. As shown in Fig. 3a, GrA at a range of concentrations suppressed the proliferation of BxPC-3 and MIA PaCa-2 cells. The IC50 values of GrA for BxPC-3 and MIA PaCa-2 cells were 0.025 μM and 0.032 μM; while those of Sal were 0.363 μM and 0.163 μM, respectively. GrA was highly potent in suppressing the proliferation of both pancreatic cancer cell lines. Compared in terms of IC50 values, GrA was more potent than Sal in the efficacy against the tested cancer cells.

The apoptosis-inducing capability of GrA was detected. As shown in Fig. 3b, GrA obviously induced early- and late-apoptosis in MIA PaCa-2 cells after treatment with 0.1 μM GrA for 24 h, around 30% cells turned to apoptosis. BxPC-3 cells were less sensitive to GrA induction of apoptosis detecting assay.

### Gramicidin A caused ultrastructural changes on cancer cell surface

Morphological changes on the surface of pancreatic cancer cells were clearly observed by SEM (Fig. 3c). As found, in the images of untreated group, there were plenty of microvilli-like protrusions all over the cell membrane surface in both of BxPC-3 and MIA PaCa-2 cells. The microvilli-like protrusions were slender and bent in appearance. After GrA treatment, the microvilli-like protrusions decreased significantly in both pancreatic cell lines. By contrast, no obvious changes were found in two cell lines treated with equal molar concentration of Sal. As shown in the images by higher magnification (Fig. 3c, right), the GrA treated cells lost almost all of the microvilli-like protrusions. In the Sal treated MIA PaCa-2 cells, the microvilli-like protrusions turned shorter and thicker; however, those of treated BxPC-3 cells remained no changes. Notably, GrA induced remarkable ultrastructural changes in the membrane of pancreatic cancer cells.

### Mitochondria structure and function were affected by gramicidin A

**Fig. 4:**
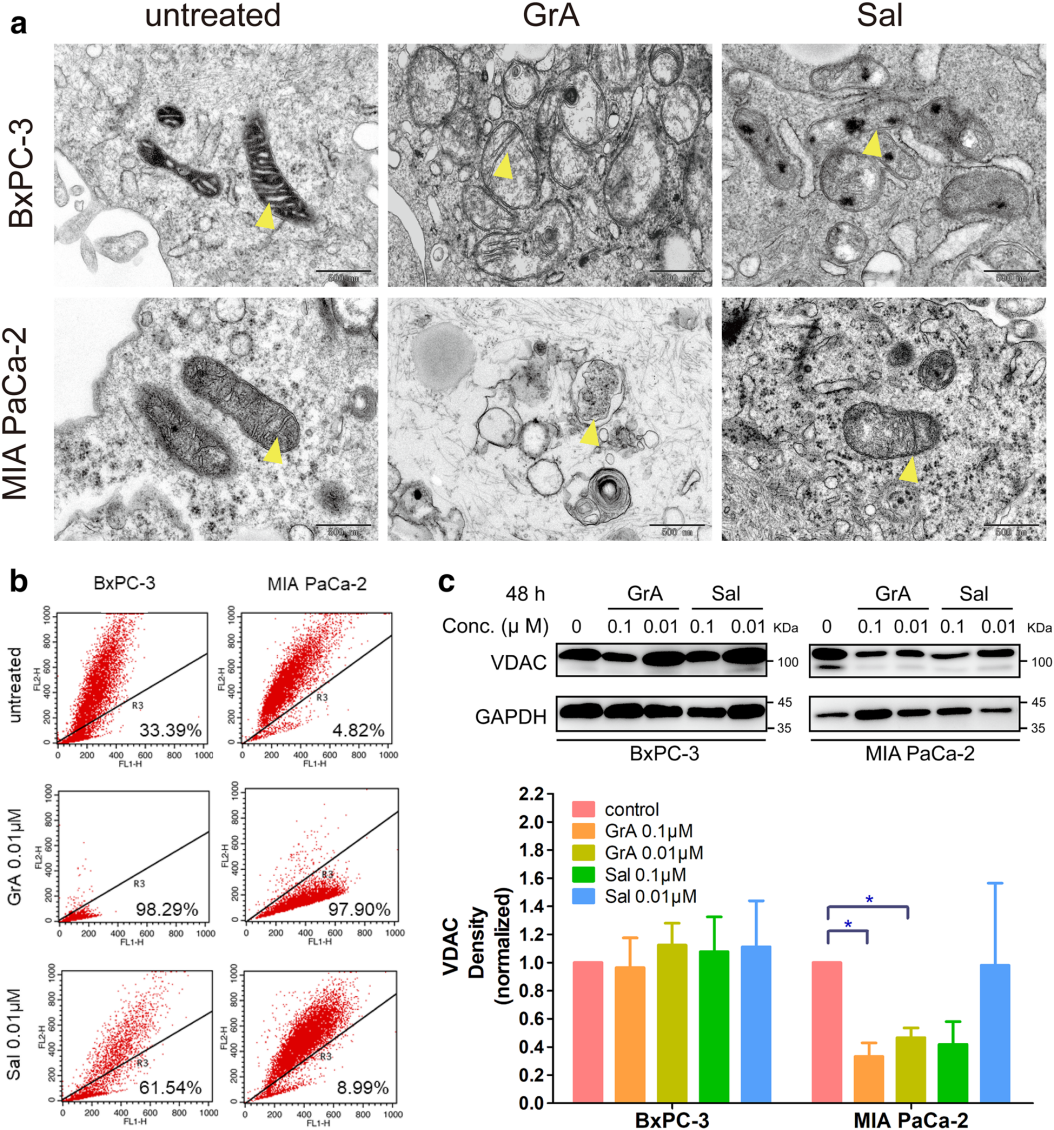
GrA altered the ultrastructure and membrane potential of mitochondria. **a** TEM images presenting morphological changes in BxPC-3 and MIA PaCa-2 cells treated with 0.05 μM GrA or Sal for 24 h. The yellow arrow indicates mitochondria in pancreatic cancer cells. Scale bar: 500 nm. **b** Effects of 0.01 μM GrA or Sal on mitochondrial membrane potential of pancreatic cells detected by flow cytometry. X-axis: green fluorescence intensity, Y-axis: red fluorescence intensity. **c** Protein expression profiles of VDAC in BxPC-3 and MIA PaCa-2 cells treated with 0.1 and 0.01 μM of GrA and Sal respectively for 48 h (upper) and its normalized density analysis (lower), *P < 0.05 in GrA vs control

Swelling of mitochondria and disappearance of cristae were found by TEM in BxPC-3 and MIAPaCa-2 cells treated with 0.05 μM GrA or Sal. More serious changes were noticed in GrA treated MIA PaCa-2 cells (not in BxPC-3 cells), showing engulfed vesicles containing aberrant mitochondria (Fig. 4a). In the experiment of detecting mitochondrial potential using JC-1 as its indicator, GrA affected mitochondrial potential much more significantly in both BxPC-3 (percentage of cells with green fluorescence: 33.39% to 98.29%, control vs GrA) and MIA PaCa-2 cells (4.82% to 97.90%) than Sal in BxPC-3 (33.39% to 61.54%, control vs Sal) and MIA PaCa-2 cells (4.82% to 8.99%) (Fig. 4b). VDAC was used as a marker to present mitochondrial function. As shown in Fig. 4c, 0.1 and 0.01 μM of GrA induced down-regulation of VDAC expression level in MIA PaCa-2 cells significantly. Sal at 0.1 μM had similar effects as GrA. The results indicated that GrA showed stronger effects than Sal on the mitochondrial functional protein at equal molar concentration.

### Gramicidin A affected the expression and distribution of pancreatic CSC markers

**Fig. 5:**
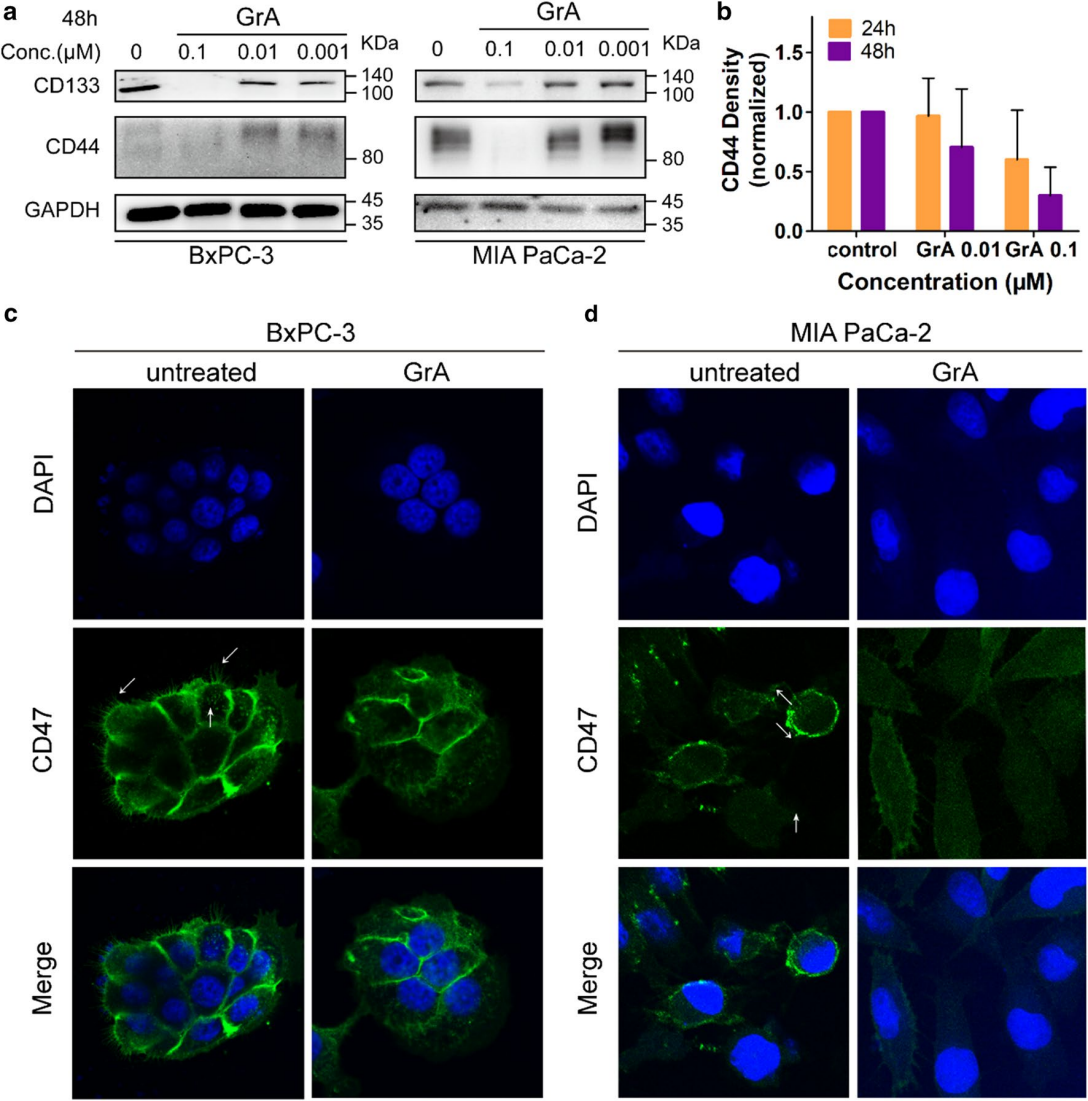
Effects of GrA on the expression and distribution of pancreatic CSC markers. **a** Protein expression profiles of CD133 and CD44 in BxPC-3 and MIA PaCa-2 cells treated with 0.1, 0.01 and 0.001 μM of GrA for 48 h represented graphically. **b** Densitometry quantitation of CD44 band intensities in western blot. Cells were treated with GrA for 24 h and 48 h, respectively. **c** Immunofluorescence staining images showed CD47 distribution in BxPC-3 cells treated with 0.05 μM GrA for 24 h. **d** Immunofluorescence staining images showed CD47 distribution in MIA PaCa-2 cells treated with 0.05 μM GrA for 24 h. Cellular nucleus was stained by DAPI exhibiting blue fluorescence while CD47 on cell surface was marked by antibodies with green fluorescence. The white arrow indicates the CD47 cluster

Pancreatic CSCs have been identified and dissected using certain cell surface antigens such as CD44 and CD133. As shown in Fig. 5a, GrA down-regulated the expression level of CD133 and CD44 in both BxPC-3 and MIA PaCa-2 cells. The summarized results of CD44 for 24 h and 48 h treatment were shown in Fig. 5b. By immunofluorescence staining, changes of cell surface CD47 distribution were captured in BxPC-3 and MIA PaCa-2 cells. In untreated BxPC-3 cells, the CD47 fluorescent signal in the cell mass border was extremely high and CD47 clusters were observed clearly. After GrA treatment the clusters turned into disordered cotton-like appearance and the border of cell mass also went dim. In untreated MIA PaCa-2 cells, bright clusters were observed in the cell surface. After GrA treatment, the CD47 clusters showed diffusive distribution in MIA PaCa-2 cells and the immunofluorescence intensity decreased on the whole surface of GrA-treated cells (Fig. 5c).

### Gramicidin A showed synergism with gemcitabine

**Fig. 6:**
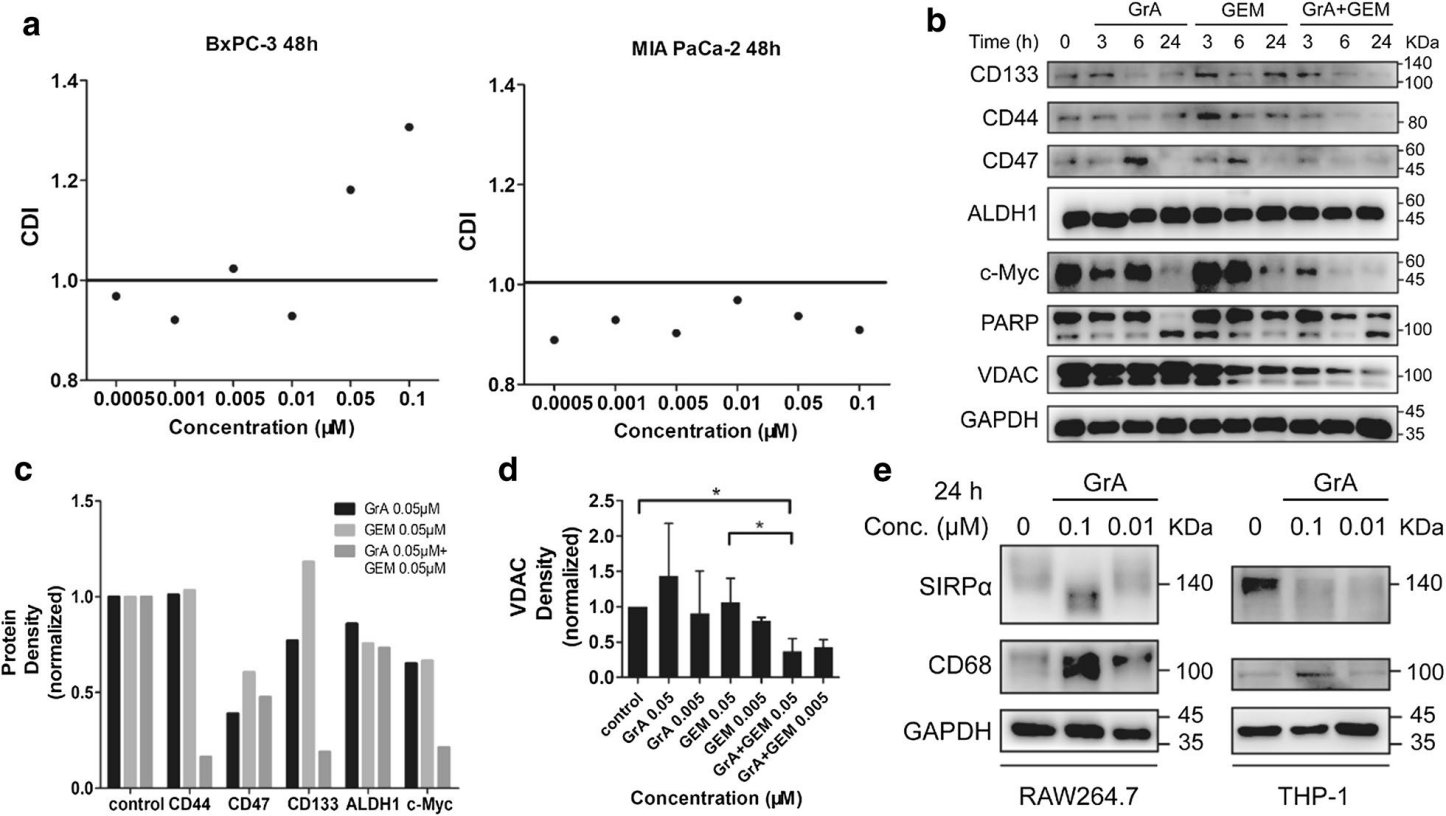
Effects of GrA and GEM combination. **a** The CDI values for GrA and GEM of BxPC-3 and MIA PaCa-2 cells were shown graphically. Viability of cultured MIA PaCa-2 cells treated with appropriate concentrations of GrA, GEM, or their combination for 48 h was determined by CCK-8 assay. Data of each concentration were from 3 replicated wells. **b** Western blot analysis of proteins from MIA PaCa-2 cells treated with 0.05 μM GrA, 0.05 μM GEM, or their combination for 3 h, 6 h, and 24 h, respectively. **c** Densitometry quantitation of 24 h treatment protein bands intensity of **b**. **d** Densitometry quantitation of VDAC band intensity from western blot experiment. Protein samples were obtained from MIA PaCa-2 cells treated with 0.05 μM, 0.005 μM GrA, GEM or their combination. *P < 0.05 in GrA + GEM 0.05 μM vs control and GrA + GEM 0.05 μM vs GEM 0.05 μM. **e** Western blot analysis of SIRPα and CD68 from RAW264.7 cells and THP-1 cells treated with 0.1 and 0.01 μM GrA

The synergistic effect of GrA with the chemotherapeutic drug GEM against cancer cells was detected by CCK-8 assay. CDI value less than 1 in two drugs combination indicates synergism. As shown, the combination of GrA and GEM exerted synergistic effect against the tested MIA PaCa-2 cells, but the two drugs synergized only in certain concentrations in BxPC-3 cells (Fig. 6a). GrA and GEM combination treatment also decreased the expression level of several CSC marker proteins in MIA PaCa-2 cells, such as CD133, CD44, CD47, and ALDH1. c-Myc protein expression level was reduced as well. Distinct PARP cleavage was observed by western blot after GrA treatment for 24 h (Fig. 6b, c). As shown in Fig. 6d, treatment of GrA combined with GEM also reduced the expression level of VDAC. The synergistic effects between GrA and GEM were demonstrated in various aspects, including the inhibition of cell proliferation and down-regulation of CSC marker expression.

The expression level of two functional proteins on macrophages were detected including SIRPα, the ligand protein of CD47, and CD68, one of the markers of tumor-associated macrophages. In both mouse macrophages cell line RAW264.7 and human monocyte cell line THP-1 (100 ng/mL PMA-activated), GrA treatment up-regulated CD68 expression level. In western blot, the band size of SIRPα in RAW264.7 cells changed from ~ 140 kDa to ~ 100 kDa and SIRPα protein in THP-1 was down-regulated (Fig. 6e).

## Discussion

GrA, a hydrophobic linear pentadecapeptide derived from microorganism, belongs to the ionophore antibiotics that some of them have been proved to target cancer stem-like cells and used as chemosensitizers [23]. Our studies have found that GrA is highly active against pancreatic CSCs as determined by tumorsphere formation assay. Compared in terms of IC50 values, the efficacy of GrA was stronger than that of Sal, the well-known CSC-active compound. GrA displayed potent cytotoxicity to pancreatic cancer cells at the levels which were much lower than the borderline concentration that could induce hemolysis. GrA induced significant morphological changes on the cell surface of cancer cells, such as the decrease or disappearance of microvilli-like protrusions. There occurred mitochondrial changes, including swelling, vacuole formation, mitochondrial potential reduction and VDAC expression down-regulation, which could cause mitochondria dysfunction that might be one of the reasons for killing cancer cells. Meanwhile, the decreased expression level of CSC markers like CD44 and CD133 indicated the percentage of CSCs may be reduced or the property of CSC stemness got weaken. The studies demonstrate that GrA is a CSC-effective agent. To our knowledge, this is the first report on the inhibitory effect of GrA against pancreatic CSCs.

Epithelial–mesenchymal transition (EMT) is a process that epithelial cells acquire the mesenchymal phenotype and mobility so that E-cadherin expression is reduced and mesenchymal markers including fibronectin and vimentin are increased. CSCs and EMT are closely related; the inducers of EMT such as TGF-β, Hedgehog or Notch could cause cells to acquire a CD44^+^ CD24^+^ ESA^+^ phenotype, which reminisces pancreatic CSCs [25]. CD44 is also considered as a mesenchymal marker in EMT [26]. MIA PaCa-2 cells with CD133 overexpression showed a more fibroblast-like morphology [25]. Reduction of CD44 and CD133 expression is correlated with loss of mesenchymal properties and acquisition of the epithelium-like phenotype in cancer cells associated with improvement of chemotherapy drug sensitivity. The interaction among EMT and CSC states and their transitions depend on context-specific factors such as intracellular, cell–cell and cell-environment signaling [27]. Emerging evidences suggest that CSCs or EMT-type cells are functionally linked. Pancreatic cancer cells surviving 6 days of continuous GEM treatment expressed higher levels of CSC markers like CD44 and CD133 along with EMT regulators like Snail and Slug [28]. Primary site tumor cells undergoing EMT turn to acquire chemoresistance and be able to suppress immune response [28–30]. PDAC is a ticklish issue with limited response to both chemotherapy and immunotherapy. Immunosuppressive nature of hypoxia in PDAC microenvironment contributes to tumor survival in many aspects, such as promoting EMT progress through pathways including NOTCH and c-MET [31]. Moreover, macrophages isolated from human PDAC were thought to induce tumor cells to produce cytidine deaminase, which metabolizes GEM to promote chemoresistance [32]. There are already clinical trials of antibody-based drug for locally advanced or metastatic pancreatic carcinoma, targeting the EMT process and its cross-link with CSCs, which may hopefully increase the survival of patients [33].

Apart from direct and fatal stimuli on cancer cell membrane system with CSC marker CD44 and CD133 expression level reduced, our studies have defined that GrA down-regulated the expression level and altered the distribution pattern of CD47. Participating in the bidirectional inhibitory signaling through both CD47 and its ligand SIRPα on macrophages [34, 35], CD47 has been regarded as the first gene known to be a target for cancer immunotherapy common to all types of cancers [36–41]. Disrupting the interaction between CD47 and SIRPα on cancer cell surface by blockade or down-regulation of CD47 efficiently promoted phagocytosis by macrophages in vitro and in vivo [42–44]. Long-term inhibition of CD47 even directly induced cancer cell apoptosis in the absence of macrophages [5]. It was previously reported that CD47 dispersed from clustering in lipid rafts into the plasma membrane in apoptosis without reduction in its expression level also resulted in loss of the high binding avidity of CD47 to SIRPα, which triggered phagocytosis [45]. In our experiment of immunofluorescence staining, the functional clustering form of CD47 on the untreated MIA PaCa-2 cell surface was significantly destroyed after GrA treatment. GrA reduced the expression level of CD47 remarkably and disrupted functional clusters of CD47 in MIA PaCa-2 cell surface, which may cause those pancreatic cancer cells recognized and engulfed by macrophages. The CD47 re-distribution on cancer cells indicated the following research about macrophage-mediated phagocytosis in CSC-targeting therapeutic approaches, leading to elimination of cancer cells in pre-clinical models [46]. On the side of macrophages, our data suggested that GrA might enhance the macrophage function as CD68 expression level increased, and down-regulate the expression level or alter the post-translational modification (such as glycosylation) on SIRPα, perhaps weakening the original anti-phagocytic signal of CD47-SIRPα. As reported, CD47 was regulated by MYC at the transcriptional level by binding promoters and the suppression of MYC through the addition of tetracycline led to the rapid down-regulation of CD47 in vitro and in vivo [47]. The present study showed that GrA synergized with GEM on decreasing the expression level of c-Myc, one of the members in MYC family and also promoting cell immortalization.

## Conclusions

In summary, the ionophore antibiotic GrA shows significant potential in suppressing pancreatic CSCs in association with CD47 down-regulation and re-distribution, implying that GrA might play a positive role in modulating the interaction between macrophages and tumor cells.

## Data Availability

All data generated and analyzed during this study are included in this published article.

## Abbreviations

CSC: cancer stem cell
GrA: gramicidin A
Sal: salinomycin
PDAC: pancreatic ductal adenocarcinoma
IC50: half-inhibitory concentration
SEM: scanning electron microscopy
TEM: transmission electron microscopy
GEM: gemcitabine
VDAC: voltage dependent anion channel
CDI: coefficient of drug interaction
Conc: concentration
PARP: poly ADP-ribose polymerase
HRP: horse radish peroxidase
RT: room temperature
PBS: phosphate buffered solution
FBS: fetal bovine serum
BSA: bovine serum albumin
TBST: tris buffered saline Tween
FITC: fluorescein isothiocyanate
PI: propidium iodide
OD: optical density
CCK-8: Cell Counting Kit 8
EMT: epithelial–mesenchymal transition
PMA: phorbol-12-myristate-13-acetate

## References

[1] Siegel R, Ma J, Zou Z, Jemal A (2014). Cancer statistics, 2014. CA Cancer J Clin.

[2] Ercan G, Karlitepe A, Ozpolat B (2017). Pancreatic cancer stem cells and therapeutic approaches. Anticancer Res.

[3] Fabian A, Vereb G, Szollosi J (2013). The hitchhikers guide to cancer stem cell theory: markers, pathways and therapy. Cytometry A.

[4] Bao Q, Zhao Y, Renner A, Niess H, Seeliger H, Jauch KW, Bruns CJ (2010). Cancer stem cells in pancreatic cancer. Cancers (Basel).

[5] Cioffi M, Trabulo S, Hidalgo M, Costello E, Greenhalf W, Erkan M, Kleeff J, Sainz B, Heeschen C (2015). Inhibition of CD47 effectively targets pancreatic cancer stem cells via dual mechanisms. Clin Cancer Res.

[6] Agliano A, Calvo A, Box C (2017). The challenge of targeting cancer stem cells to halt metastasis. Semin Cancer Biol.

[7] Keysar SB, Jimeno A (2010). More than markers: biological significance of cancer stem cell-defining molecules. Mol Cancer Ther.

[8] Ablett MP, Singh JK, Clarke RB (2012). Stem cells in breast tumours: are they ready for the clinic?. Eur J Cancer.

[9] Song IS, Jeong JY, Jeong SH, Kim HK, Ko KS, Rhee BD, Kim N, Han J (2015). Mitochondria as therapeutic targets for cancer stem cells. World J Stem Cells.

[10] Laws K, Bineva-Todd G, Eskandari A, Lu C, O’Reilly N, Suntharalingam K (2018). A copper(II) phenanthroline metallopeptide that targets and disrupts mitochondrial function in breast cancer stem cells. Angew Chem Int Ed Engl.

[11] Rausch V, Liu L, Kallifatidis G, Baumann B, Mattern J, Gladkich J, Wirth T, Schemmer P, Buchler MW, Zoller M (2010). Synergistic activity of sorafenib and sulforaphane abolishes pancreatic cancer stem cell characteristics. Cancer Res.

[12] Bao B, Wang Z, Ali S, Ahmad A, Azmi AS, Sarkar SH, Banerjee S, Kong D, Li Y, Thakur S (2012). Metformin inhibits cell proliferation, migration and invasion by attenuating CSC function mediated by deregulating miRNAs in pancreatic cancer cells. Cancer Prev Res (Phila).

[13] Zhang GN, Liang Y, Zhou LJ, Chen SP, Chen G, Zhang TP, Kang T, Zhao YP (2011). Combination of salinomycin and gemcitabine eliminates pancreatic cancer cells. Cancer Lett.

[14] Kelkar DA, Chattopadhyay A (2007). The gramicidin ion channel: a model membrane protein. Biochim Biophys Acta.

[15] David JM, Rajasekaran AK (2015). Gramicidin A: a new mission for an old antibiotic. J Kidney Cancer VHL.

[16] Naujokat C, Steinhart R (2012). Salinomycin as a drug for targeting human cancer stem cells. J Biomed Biotechnol.

[17] Choi YJ, Gurunathan S, Kim JH (2018). Graphene oxide-silver nanocomposite enhances cytotoxic and apoptotic potential of salinomycin in human ovarian cancer stem cells (OvCSCs): a novel approach for cancer therapy. Int J Mol Sci.

[18] Gupta PB, Onder TT, Jiang G, Tao K, Kuperwasser C, Weinberg RA, Lander ES (2009). Identification of selective inhibitors of cancer stem cells by high-throughput screening. Cell.

[19] Rao DK, Liu H, Ambudkar SV, Mayer M (2014). A combination of curcumin with either gramicidin or ouabain selectively kills cells that express the multidrug resistance-linked ABCG2 transporter. J Biol Chem.

[20] David JM, Owens TA, Barwe SP, Rajasekaran AK (2013). Gramicidin A induces metabolic dysfunction and energy depletion leading to cell death in renal cell carcinoma cells. Mol Cancer Ther.

[21] David JM, Owens TA, Inge LJ, Bremner RM, Rajasekaran AK (2014). Gramicidin A blocks tumor growth and angiogenesis through inhibition of hypoxia-inducible factor in renal cell carcinoma. Mol Cancer Ther.

[22] Chakraborty K, Dutta C, Mukherjee S, Biswas A, Gayen P, George G, Raghothama S, Ghosh S, Dey S, Bhattacharyya D (2018). Engineering ionophore gramicidin-inspired self-assembled peptides for drug delivery and cancer nanotherapeutics. Adv Ther.

[23] Kaushik V, Yakisich JS, Kumar A, Azad N, Iyer AKV (2018). Ionophores: potential use as anticancer drugs and chemosensitizers. Cancers (Basel).

[24] Zheng YB, Gong JH, Liu XJ, Li Y, Zhen YS (2017). A CD13-targeting peptide integrated protein inhibits human liver cancer growth by killing cancer stem cells and suppressing angiogenesis. Mol Carcinog.

[25] Zhou P, Li B, Liu F, Zhang M, Wang Q, Liu Y, Yao Y, Li D (2017). The epithelial to mesenchymal transition (EMT) and cancer stem cells: implication for treatment resistance in pancreatic cancer. Mol Cancer.

[26] Chen T, You Y, Jiang H, Wang ZZ (2017). Epithelial–mesenchymal transition (EMT): a biological process in the development, stem cell differentiation, and tumorigenesis. J Cell Physiol.

[27] Burger GA, Danen EHJ, Beltman JB (2017). Deciphering epithelial–mesenchymal transition regulatory networks in cancer through computational approaches. Front Oncol.

[28] Quint K, Tonigold M, Di Fazio P, Montalbano R, Lingelbach S, Ruckert F, Alinger B, Ocker M, Neureiter D (2012). Pancreatic cancer cells surviving gemcitabine treatment express markers of stem cell differentiation and epithelial–mesenchymal transition. Int J Oncol.

[29] Gloushankova NA, Zhitnyak IY, Rubtsova SN (2018). Role of epithelial–mesenchymal transition in tumor progression. Biochemistry (Mosc).

[30] Yin T, Wei H, Gou S, Shi P, Yang Z, Zhao G, Wang C (2011). Cancer stem-like cells enriched in Panc-1 spheres possess increased migration ability and resistance to gemcitabine. Int J Mol Sci.

[31] Daniel SK, Sullivan KM, Labadie KP, Pillarisetty VG (2019). Hypoxia as a barrier to immunotherapy in pancreatic adenocarcinoma. Clin Transl Med.

[32] Arnold JN, Magiera L, Kraman M, Fearon DT (2014). Tumoral immune suppression by macrophages expressing fibroblast activation protein-alpha and heme oxygenase-1. Cancer Immunol Res.

[33] Beuran M, Negoi I, Paun S, Ion AD, Bleotu C, Negoi RI, Hostiuc S (2015). The epithelial to mesenchymal transition in pancreatic cancer: a systematic review. Pancreatology.

[34] Barclay AN, Brown MH (2006). The SIRP family of receptors and immune regulation. Nat Rev Immunol.

[35] Barclay AN (2009). Signal regulatory protein alpha (SIRPalpha)/CD47 interaction and function. Curr Opin Immunol.

[36] Zhang M, Hutter G, Kahn SA, Azad TD, Gholamin S, Xu CY, Liu J, Achrol AS, Richard C, Sommerkamp P (2016). Anti-CD47 treatment stimulates phagocytosis of glioblastoma by M1 and M2 polarized macrophages and promotes M1 polarized macrophages in vivo. PLoS ONE.

[37] Ngo M, Han A, Lakatos A, Sahoo D, Hachey SJ, Weiskopf K, Beck AH, Weissman IL, Boiko AD (2016). Antibody therapy targeting CD47 and CD271 effectively suppresses melanoma metastasis in patient-derived xenografts. Cell Rep.

[38] Weiskopf K, Jahchan NS, Schnorr PJ, Cristea S, Ring AM, Maute RL, Volkmer AK, Volkmer JP, Liu J, Lim JS (2016). CD47-blocking immunotherapies stimulate macrophage-mediated destruction of small-cell lung cancer. J Clin Invest.

[39] Weiskopf K (2017). Cancer immunotherapy targeting the CD47/SIRPalpha axis. Eur J Cancer.

[40] Tseng D, Volkmer JP, Willingham SB, Contreras-Trujillo H, Fathman JW, Fernhoff NB, Seita J, Inlay MA, Weiskopf K, Miyanishi M (2013). Anti-CD47 antibody-mediated phagocytosis of cancer by macrophages primes an effective antitumor T-cell response. Proc Natl Acad Sci USA.

[41] Weissman I (2016). How one thing led to another. Annu Rev Immunol.

[42] Willingham SB, Volkmer J-P, Gentles AJ, Sahoo D, Dalerba P, Mitra SS, Wang J, Contreras-Trujillo H, Martin R, Cohen JD (2012). The CD47-signal regulatory protein alpha (SIRPa) interaction is a therapeutic target for human solid tumors. Proc Natl Acad Sci.

[43] Liu J, Wang L, Zhao F, Tseng S, Narayanan C, Shura L, Willingham S, Howard M, Prohaska S, Volkmer J (2015). Pre-clinical development of a humanized anti-CD47 antibody with anti-cancer therapeutic potential. PLoS ONE.

[44] Chao MP, Alizadeh AA, Tang C, Myklebust JH, Varghese B, Gill S, Jan M, Cha AC, Chan CK, Tan BT (2010). Anti-CD47 antibody synergizes with rituximab to promote phagocytosis and eradicate non-Hodgkin lymphoma. Cell.

[45] Lv Z, Bian Z, Shi L, Niu S, Ha B, Tremblay A, Li L, Zhang X, Paluszynski J, Liu M, Zen K, Liu Y (2015). Loss of cell surface CD47 clustering formation and binding avidity to SIRPα facilitate apoptotic cell clearance by macrophages. J Immunol.

[46] Chao MP, Weissman IL, Majeti R (2012). The CD47-SIRPalpha pathway in cancer immune evasion and potential therapeutic implications. Curr Opin Immunol.

[47] Casey SC, Tong L, Li Y, Do R, Walz S, Fitzgerald KN, Gouw AM, Baylot V, Gutgemann I, Eilers M (2016). MYC regulates the antitumor immune response through CD47 and PD-L1. Science.

